# Is *Demodex* hiding in lichen planopilaris pathogenesis? The trichoscopy-blind zone

**DOI:** 10.1093/skinhd/vzag015

**Published:** 2026-03-20

**Authors:** Kambiz Kamyab Hesari, Alireza Ghanadan, Vahidehsadat Azhari, Sahar Montazeri, Haleh Tavakolian, Mina Saber

**Affiliations:** Department of Dermatopathology, Razi Hospital, Tehran University of Medical Sciences, Tehran, Iran; Department of Dermatopathology, Razi Hospital, Tehran University of Medical Sciences, Tehran, Iran; Department of Dermatopathology, Razi Hospital, Tehran University of Medical Sciences, Tehran, Iran; Department of Dermatopathology, Razi Hospital, Tehran University of Medical Sciences, Tehran, Iran; Department of Dermatopathology, Razi Hospital, Tehran University of Medical Sciences, Tehran, Iran; Department of Dermatology, Skin Diseases and Leishmaniasis Research Center, School of Medicine, Isfahan University of Medical Sciences, Isfahan, Iran

## Abstract

The pathogenic role of commensal *Demodex* mites in lichen planopilaris remains hypothetical. We propose a multifactorial model where mite lipases and antigens disrupt follicular peroxisome proliferator-activated receptor gamma (PPAR-γ) signalling and trigger a lymphocytic response that may progress to autoimmunity. A therapeutic paradox exists, as immunomodulatory treatments may reduce inflammation but also promote mite proliferation, underscoring the need for controlled trials.

Dear Editor, *Demodex* mites are harmless organisms that live in human hair follicles. *Demodex folliculorum* primarily resides in the follicular infundibulum, whereas *Demodex brevis* is located deeper within sebaceous glands, breaking down sebum with lipase secretion.^1^ Although colonization rates in healthy individuals vary widely from 10% to 80%, their role in human skin remains debated. Some researchers propose a symbiotic relationship, suggesting that *Demodex* might help control follicular bacteria. However, growing evidence suggests that these mites can act as pathogens in certain skin conditions, including rosacea, chronic blepharitis, perioral dermatitis and various types of alopecia.^2^ Scalp demodicosis is a rarely recognized condition with only a few reported cases, presenting as patchy hair loss, scalp redness, scaling and pustules.^3^ Recent observations suggest a possible association between *Demodex* infestation and lichen planopilaris (LPP), a primary cicatricial alopecia characterized by perifollicular inflammation and fibrosis, but the clinical relevance of this relationship remains unclear. Several lines of evidence raised interest in this association (Figure 1).

**Figure 1:**
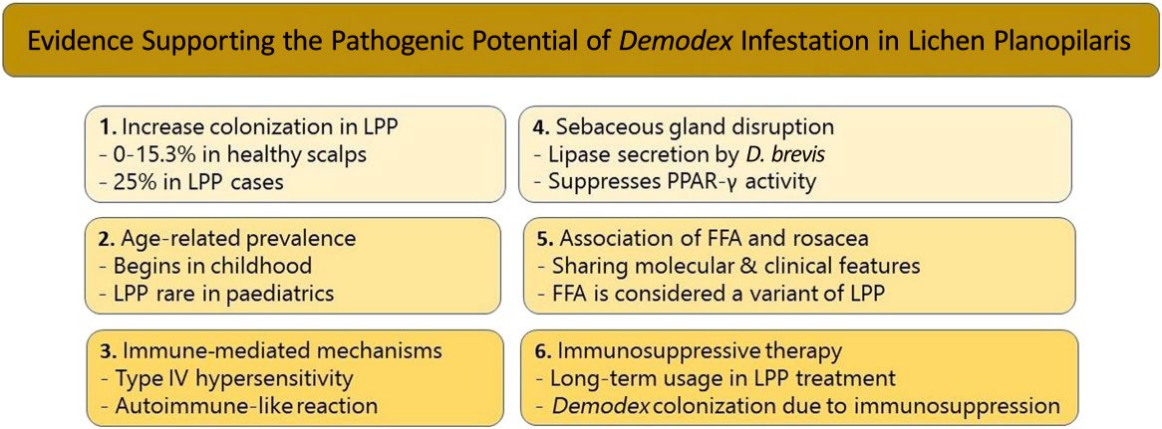
Overview of *Demodex*-related mechanisms in lichen planopilaris (LPP). FFA, frontal fibrosing alopecia; PPAR, peroxisome proliferator-activated receptor.

In a healthy scalp, the *Demodex* colonization rate ranges from 0% to 15.3%.^3^ However, a histopathological study found *Demodex* mites in 25% of LPP cases,^4^ suggesting a possible pathogenic role. *Demodex* colonization usually begins in childhood or early adulthood,^3^ which coincides with the rarity of LPP in children, indicating that long-term mite presence may contribute to chronic follicular inflammation. The mites’ antigens trigger a type IV hypersensitivity reaction, leading to spongiosis and perifollicular lymphocytic infiltration.^2^ This inflammation can expose hidden follicular antigens, potentially activating an autoimmune response similar to LPP. Furthermore, *D. brevis* resides deep within sebaceous glands, secreting lipase and disrupting skin lipid composition. As follicular peroxisome proliferator-activated receptor gamma (PPAR-γ) dysfunction has been implicated in LPP pathogenesis, ­mite-induced changes in sebaceous glands might potentially affect PPAR-γ activity and could play a role in disease progression.^1,2^

Additionally, frontal fibrosing alopecia (FFA) and rosacea are connected clinically through gene expression profiles and underlying molecular mechanisms.^5^ Although a link between *Demodex* colonization and rosacea has been confirmed, it remains speculative whether a similar relationship may exist between *Demodex* and FFA, a variant of LPP. A major concern in treating LPP and FFA is the potential unintended increase in *Demodex* colonization caused by immunosuppressive therapies. Topical corticosteroids, calcineurin inhibitors and systemic immunomodulators can potentially weaken local immunity, enabling mite overgrowth.^3^ This may create a paradox where anti-inflammatory treatments, while reducing LPP-related inflammation, may also encourage *Demodex*-driven pathology. Altemir *et al*. reported that FFA-like inflammation in patients with *Demodex* overgrowth resolved after stopping topical steroids and administering ivermectin.^6^ These findings suggest that *Demodex* infestation may exacerbate or mimic LPP/FFA inflammation, particularly in patients who are immunosuppressed.

To evaluate *Demodex* colonization in the scalp of patients with LPP, we retrospectively analysed trichoscopic images of 150 individuals with active LPP from our alopecia clinic database, specifically noting the presence of the ‘*Demodex* tail’ sign (a characteristic trichoscopic feature of superficial *Demodex* infestation^7^). Interestingly, none of the cases showed this sign. While this may indicate the absence of superficial *D. folliculorum*, it raises the possibility that *D. brevis*, which lives deeper within follicular structures, may still play a role in the development of LPP by affecting lipid metabolism and PPAR-γ signalling, potentially escaping routine trichoscopic detection.

Among these patients, pathology slides were available for 98 individuals, with an average of 15–18 sections per case. Review revealed *Demodex* infestation in 19 patients (19%). Of these positive cases, one had *D. folliculorum*, while the remaining 18 tested positive for *D. brevis* (Figure 2). Notably, exhaustive processing of all tissue levels was not performed, so the true prevalence of *Demodex* infestation may be underestimated. Processing all tissue samples could reasonably lead to an increased detection rate. Additionally, drug-induced local immunosuppression probably increases the chances of *Demodex* proliferation in treated patients. Therefore, if samples were obtained from patients who were undertreated, the positive rate might be higher. Based on the available evidence, a hypothesis can be generated that *Demodex* mites, particularly *D. brevis*, may contribute to the pathogenesis of LPP, as either initial triggers or secondary aggravators in individuals who are immunosuppressed. Given the often-refractory nature of LPP to current therapeutic options, the investigation of anti-*Demodex* agents, such as ivermectin, in treatment-resistant cases may warrant consideration. This hypothesis requires verification through further research, ideally including case–control studies and controlled clinical trials that correlate clinical outcomes with quantitative assessments of mite density before and after intervention. Such studies would be valuable for clarifying the potential role of these ectoparasites and for refining the management of this challenging cicatricial alopecia.

**Figure 2:**
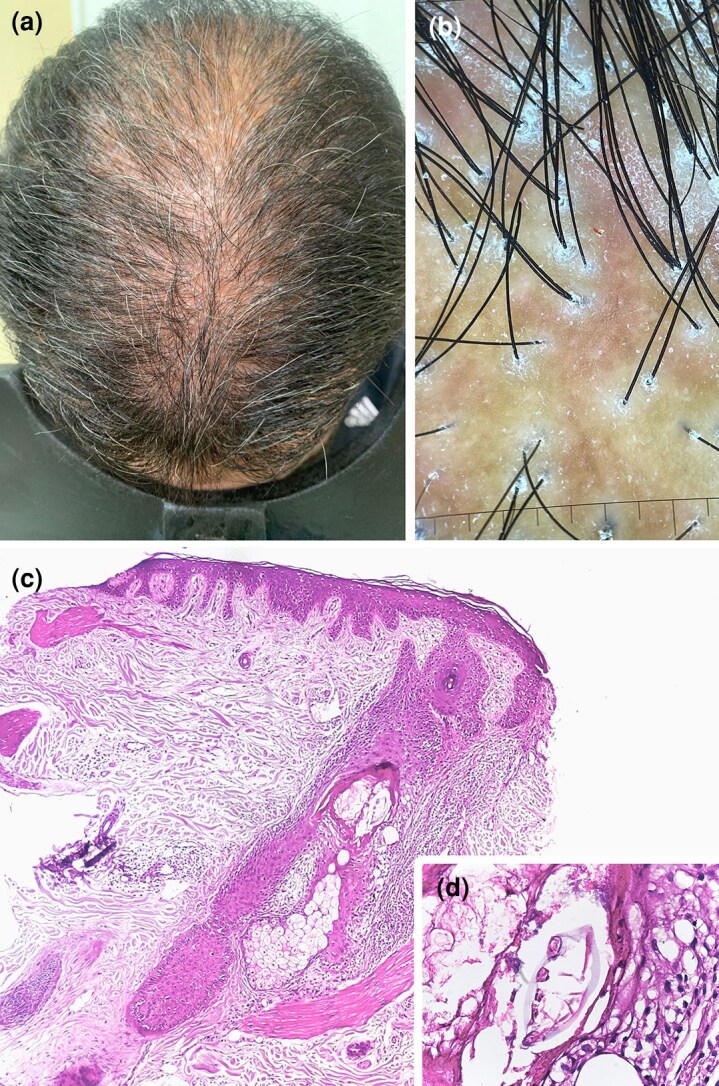
Histopathological examination (haematoxylin and eosin stain) of scalp skin shows a hair follicle containing *Demodex brevis* mite. There is a prominent lichenoid perifollicular lymphocytic infiltrate, mainly composed of small lymphocytes around the infundibular and isthmic parts of the follicle. Mild interface changes and perifollicular fibrosis are observed (a, ×20). *Demodex brevis* is present in the sebaceous canal (b, ×100).

In summary, considering the points outlined above, it can be hypothesized that *Demodex* infestation, particularly *D. brevis*, may be associated with the development or exacerbation of patients with LPP, especially those who are receiving immunosuppressive treatment. Both systemic and topical immunosuppression may potentially create conditions that favour *Demodex* proliferation. These observations are hypothesis generating, and further well-designed studies are required to clarify the nature and significance of this possible association.

## Data Availability

The data underlying this article will be shared on reasonable request to the corresponding author.
